# Building governance reform without accountability transfer invites urban disaster

**DOI:** 10.1038/s41467-026-78062-2

**Published:** 2026-09-24

**Authors:** Yasemin D. Aktas, Cassidy Johnson

**Affiliations:** 1 Department of Civil, Environmental and Geomatic Engineering, University College London (UCL), London, UK; 2 The Bartlett Development Planning Unit, University College London (UCL), London, UK

**Keywords:** Government, Natural hazards

## Abstract

Building governance reforms can displace accountability rather than transfer it. The 2023 Türkiye earthquake disaster – in which formally inspected, post-reform buildings collapsed at scale – exemplifies this failure mode and reveals the institutional conditions required for genuine accountability transfer.

## A general failure mode of building governance

When the 2023 Kahramanmaraş earthquake sequence killed more than 50,000 people and destroyed or severely damaged over 518,000 buildings across eleven provinces of Türkiye, the disaster was widely attributed to corruption and negligence^1^. This framing, while not inaccurate, is insufficient: it locates the failure in individuals when the failure is systemic. A disproportionate share of the buildings that collapsed had been formally inspected and built after a comprehensive programme of regulatory reform^2^. This is not primarily a story about individual wrongdoing. It is a story about the architecture of governance.

We term the underlying condition *accountability displacement*: a governance state in which reform activity is high, nominal compliance is achievable, and yet no actor bears traceable responsibility for outcomes. The concept does not presuppose a prior state of functioning accountability; rather, it names the condition in which the formal architecture of accountability—inspectors, permits, sign-offs, legislative oversight—is present and internally coherent, while the functional capacity to hold responsible actors to account is systematically absent. Three features define it: the formal appearance of a functioning accountability chain; the systematic removal of mechanisms by which individuals can be held responsible for specific failures; and the periodic collective absorption of liability by the state through disaster response, reconstruction, or legislative amnesty. Accountability displacement differs from simple corruption—it is compatible with actors behaving legally at every stage, while the governance system as a whole produces unaccountable outcomes.

Türkiye offers the most data-rich contemporary demonstration of this mechanism. But accountability displacement is not unique to Türkiye, nor to seismic risk. It is a general failure mode of building governance systems in rapidly urbanising contexts, and understanding it is a prerequisite for designing reforms capable of achieving genuine accountability transfer.

## Türkiye: a fully documented case

The 1999 Marmara earthquakes triggered Türkiye’s most significant building governance overhaul^3^. Responsibility for building safety in Türkiye is distributed across four main actors. The Ministry of Environment, Urbanisation and Climate Change (MoEUCC) sets building codes, licenses inspection firms, and manages the national inspection database. Municipalities issue building permits and conduct final occupancy sign-offs. Licensed private inspection firms, operating under Law 4708 (2001), certify construction during the build process. The Grand National Assembly enacts the legislative framework, including the construction amnesties discussed below.

What this structure lacks is a continuous accountability chain connecting these actors to specific outcomes.

**Fig. 1:**
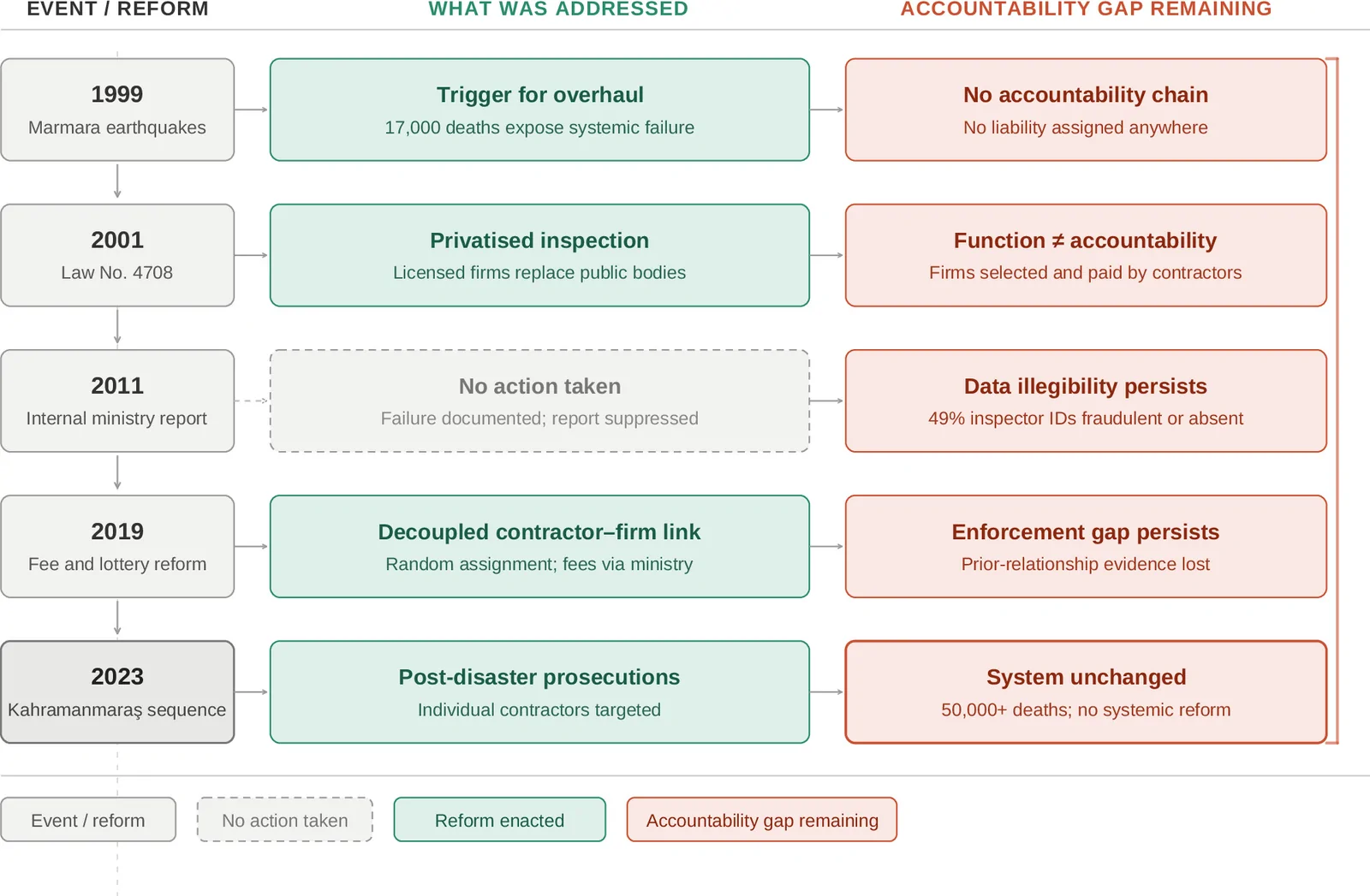
Reform without accountability transfer: Türkiye’s building inspection governance, 1999–2023. Each row shows what a reform nominally addressed (green) and the accountability gap that persisted (coral). The 2011 ministry report documented systemic failure but produced no action (dashed grey). The coral column remains unbroken across all five rows, illustrating the pattern we term accountability displacement: reform activity high, accountability structure unchanged.

The 2001 Building Inspection Law privatised inspection, creating licensed firms operating under nominal ministry supervision – a market-based logic that assumed competition would produce rigour^4,5^. It did not: firms were paid by building owners and selected by contractors, systematically incentivising permissiveness^6^. Engineers rented out credentials without conducting site visits^7^. A 2011 internal ministry audit documented the absence of meaningful oversight – and was not acted upon^2^. A further round of reforms in 2019 severed the direct financial link between contractors and inspection firms through a lottery assignment system, but left three governance failures intact (Fig. 1).

The first governance failure is that the inspection database produces illegibility rather than accountability: a 2024 investigation by the Turkish service of the BBC found that 49% of inspector ID information was inaccurate or fraudulent, 66% of technical personnel records were wrong, and records from 2008–2011 exist in no backup form^2^. In the aftermath of the 2023 earthquakes, these data gaps made it difficult in many cases to reliably establish which firm or inspector was responsible for a specific building, weakening the evidentiary basis for holding identified actors to account. Second, inspection engineers are paid below the poverty line in salary terms, and without the requirement to have a seismic or even civil engineering specialisation: an electronics engineer is legally permitted to serve as site engineer on a high-rise in a seismic zone^2,7^. Third, since 1948, Türkiye has enacted twenty construction amnesties—legislative instruments that retroactively legalise non-compliant buildings, systematically privatising the gains from illegal construction while benefitting ruling parties during elections and socialising the long-term losses through state-funded disaster response^8,9^. Post-disaster reconstruction can compound this dynamic, as the speed and scale of state-led housing programmes risk reproducing the same accountability gaps under conditions of urgency that override legibility.

The theoretical significance of accountability displacement lies in its capacity to explain why reform produces persistence of failure. If accountability is displaced rather than absent, adding more rules, more inspectors, or more competition does not address the underlying condition—and may deepen it. The 2019 reform which introduced the lottery is instructive: by randomising firm assignment, it removed one visible form of corruption—the prior commercial relationship between contractor and inspection firm that could constitute evidence of collusion—yet it left the underlying accountability framework intact^10^. Attribution of responsibility to specific inspectors and firms is possible in principle, and formal penalties exist for identified actors – but attribution does not translate into enforceable accountability: identifying a responsible party is not the same as securing a consequence against them. This gap between formal attribution and enforceable accountability is a separate and prior failure from the lottery reform: it stems from the combination of data illegibility and the difficulty of securing enforceable outcomes documented above, and the lottery mechanism does not address either.

## Beyond Türkiye: a comparative pattern

The privatisation of building control functions has been adopted across a range of countries following governance reform agendas that emphasise market mechanisms and reduced state bureaucracy^11^. The inherent tension this creates—that private inspection generates accountability to the client rather than the public—has been noted in comparative building control literature but not theorised in relation to disaster outcomes^11,12^.

Each element of Türkiye’s accountability displacement is observable in different geographies, though in different combinations and with different emphases across contexts. No single case replicates the full syndrome, but what the comparative record shows is that the components are not uniquely Turkish.

The amnesty mechanism is the most widely documented. Italy’s *condono edilizio* system has processed more than fifteen million applications to regularise illegal construction since 1985, many in seismically active zones^13^. The consequences extend beyond the building stock: empirical analysis of the 1976 Friuli and 1980 Irpinia earthquakes—comparable in magnitude but occurring in regions with markedly different institutional quality—shows that where governance was weaker and regularisation of non-compliant construction more entrenched, post-disaster economic recovery was markedly slower^14^. A synthetic control design accounts for pre-existing differences in economic trajectories between the two regions. The relevance for our argument is that amnesties do more than legalise unsafe buildings: by entrenching a governance environment in which non-compliance carries no consequence, they weaken the institutional capacity on which recovery itself depends. In Peru and Ecuador, large-scale programmes to regularise informal settlements in seismically active cities have proceeded without seismic risk assessment as a precondition for legalisation^15,16^, illustrating how the amnesty mechanism operates independently of whether a formal inspection system exists at all.

The tension in privatised inspection—that private firms are accountable to the client rather than to the public—is not unique to Türkiye. Van der Heijden’s comparative analysis of building regulatory enforcement in Australia, Canada and the Netherlands documents the same dynamic across high-income contexts^11^: where inspection is market-provided, competitive pressure systematically favours permissiveness over rigour, regardless of formal oversight structures^12^.

The data illegibility problem is harder to evidence comparatively, and Türkiye’s documented case—fraudulent identifiers, missing records, an unrecoverable four-year gap in its records (2008–2011)—may be unusually severe. The impact of Nepal’s 2015 Gorkha earthquake points to an analogous condition: in rapidly urbanising areas outside Kathmandu, over 80% of buildings were found not to comply with the post-1988 National Building Code^17,18^. This implies an enforcement and recording system incapable of producing the attribution trails that post-disaster justice requires, even if the specific data infrastructure failures have not been documented to the same degree.

## What accountability transfer requires

Three conditions must be met simultaneously for accountability transfer to occur. First, data legibility must be treated as a governance priority: building records must be integrated with national identity systems, immutable after submission, and sufficient to support post-hoc attribution of professional responsibility. A building inspection system that cannot identify or hold to account those who certified a specific building is not an inspection system; it is a document-generation system with no deterrent function.

Second, liability must be explicitly assigned and structurally maintained. Privatised inspection is not inherently incompatible with accountability, but requires that liability be explicitly assigned and institutionally maintained across the full construction chain—not extinguished by sale of the building or the passage of time. Türkiye’s own post-1999 reform proposals included exactly this mechanism: inspection firm liability insurance was proposed but never enacted, partly because the insurance industry would not underwrite a sector whose data structures could not support actuarial assessment^19^. Chile’s post-2010 model offers a working precedent: statutory liability runs from geotechnical and structural engineers through developers for the latter up to ten years after completion and covers all architectural and structural components, all responsible professionals and their interactions during construction are named in a publicly accessible construction record. Independent seismic peer review has been mandatory since 2003—creating the traceable accountability chain that Türkiye’s system lacks^20^. Individual professional liability is one component of this model, not its totality: the Chilean system works because individual accountability is embedded within a broader state-maintained framework of records, mandatory seismic code, material and site inspections, peer review, and public transparency. The lesson is not that engineers alone should bear risk, but that the state must design and maintain the infrastructure through which professional accountability can be meaningfully exercised and enforced.

Third, *construction amnesties in seismically active zones must be subject to mandatory hazard assessment* before enactment. Amnesties conditioned on seismic risk evaluation transform the instrument from a liability transfer mechanism into a managed compliance pathway. This principle must extend to post-disaster reconstruction: emergency housing programmes that bypass normal permitting and inspection processes in the name of speed risk reproducing the very accountability voids they follow. Reconstruction amnesties—whether formal or de facto—should be subject to the same hazard assessment requirement. The Sendai Framework’s call for accountability for disaster risk creation^21^ remains unoperationalised in relation to building amnesties, including those enacted in reconstruction contexts, in all current implementation guidance.

Türkiye trains some of the world’s finest earthquake engineers and operates technically sophisticated seismic codes. The 2023 disaster cannot be attributed to ignorance. What it exposes is a governance gap that technical excellence cannot bridge: between knowing what safe construction requires and building a system in which the actors responsible for delivering it are reliably accountable for doing so. That gap—and the political conditions that sustain it—is not a Türkiye-specific pathology. It demands collaborative attention from engineers, planners, governance scholars, and policymakers alike—each of whom holds part of the solution that no single discipline can supply alone.
